# Controversial Past, Splendid Present, Unpredictable Future: A Brief Review of Alzheimer Disease History

**DOI:** 10.3390/jcm13020536

**Published:** 2024-01-17

**Authors:** Félix Bermejo-Pareja, Teodoro del Ser

**Affiliations:** 1 CIBERNED, Institute of Health Carlos III, 28029 Madrid, Spain; 2 Institute of Research i+12, University Hospital “12 de Octubre”, 28041 Madrid, Spain; 3 Alzheimer’s Centre Reina Sofia—CIEN Foundation, Institute of Health Carlos III, 28031 Madrid, Spain; tdelser@fundacioncien.es

**Keywords:** Alzheimer disease, elderly dementia, past history, future, nosological concept, hypothesis, pathology, physiopathology, therapy, prevention

## Abstract

**Background:** The concept of Alzheimer disease (AD)—since its histological discovery by Alzheimer to the present day—has undergone substantial modifications. **Methods:** We conducted a classical narrative review of this field with a bibliography selection (giving preference to Medline best match). **Results:** The following subjects are reviewed and discussed: Alzheimer’s discovery, Kraepelin’s creation of a new disease that was a rare condition until the 1970′s, the growing interest and investment in AD as a major killer in a society with a large elderly population in the second half of the 20th century, the consolidation of the AD clinicopathological model, and the modern AD nosology based on the dominant amyloid hypothesis among many others. In the 21st century, the development of AD biomarkers has supported a novel biological definition of AD, although the proposed therapies have failed to cure this disease. The incidence of dementia/AD has shown a decrease in affluent countries (possibly due to control of risk factors), and mixed dementia has been established as the most frequent etiology in the oldest old. **Conclusions:** The current concept of AD lacks unanimity. Many hypotheses attempt to explain its complex physiopathology entwined with aging, and the dominant amyloid cascade has yielded poor therapeutic results. The reduction in the incidence of dementia/AD appears promising but it should be confirmed in the future. A reevaluation of the AD concept is also necessary.

*Et très peu d’histoire… vaut mieux que pas d’histoire du tout.*

Claude Lévi-Strauss

## 1. Introduction

This review outlines the complex evolution of Alzheimer disease (AD) from Alois Alzheimer’s initial histological discovery in the early 20th century to the present day. Among chronic neurological disorders, AD is likely the most intriguing, having undergone the greatest conceptual changes throughout its history. Furthermore, the future evolution of our understanding of AD remains an ongoing open process.

For an adequate historical perspective, it is important to recall that age-related cognitive decline has been recognized since the times of Ancient Egypt and has been referred to by various terms [1,2,3]. The current term, dementia, has a Latin root meaning “without mind” [1,2,3]. Greek thinkers, such as Pythagoras and Hippocrates, postulated a brain origin for this disorder primarily caused by “senility” [4], and the Greco-Roman medical doctrine was analogous, attributing the irreversible deterioration of mental capacities in old age to senility [3,4,5]. Notwithstanding, Cicero thought that dementia was not always a consequence of old age or senility, but rather that an active mental life could prevent or lessen it, and that frequently human knowledge may increase in old age [6].

The term *dementia* began its medical usage in the 19th century and was fueled by French neurologists like Pinel and Esquirol [1,3]. In the last third of that century, the discoveries of brain alterations in various mental disorders (such as syphilitic general paresis), alongside new brain fixation and histological staining techniques enabled better study of brain tissue and established a neuropathological foundation for dementia research in the 20th century [3,7]. Blocq and Marinesco first described in 1892 in an epileptic patient the lesions that would later be known as *senile plaques* (SP) [8], and Redlich published in 1898 a detailed description of SP in a case of senile dementia (SD) [9]. The description of this histological marker of AD and other SD was the first milestone in the history of this dementia disorder whose evolution during the 20th and 21th centuries is the objective of this review.

## 2. Methods

This review is an authors’ classical narrative review of a medical subject. To review the immense amount of literature about AD in the last thirty years is challenging. To overcome this difficulty, we selected the most important and recent articles of each relevant author in the dementia/AD field (psychiatry, neurology, basic sciences). We explored Medline (National Library of Medicine, Bethesda, MD, USA) the most complete database for clinical biomedicine, using the *best match* approach [10], and Google Academic (Mountain View, CA, USA), the wider medical database, to obtain the most relevant and recent articles. Both authors agreed on the selection of references in this review. We also took historical texts on this subject into account along with our own memories of meetings and personal contacts with several leaders in this field. This historical review is mainly made from the perspective of two clinical neurologists, although some borderline social aspects are briefly discussed. 

## 3. Narrative Review: Dementia/AD during 20th and 21th Centuries

### 3.1. The Alzheimer’s Discovery: A Controversial Past

**Figure 1:**
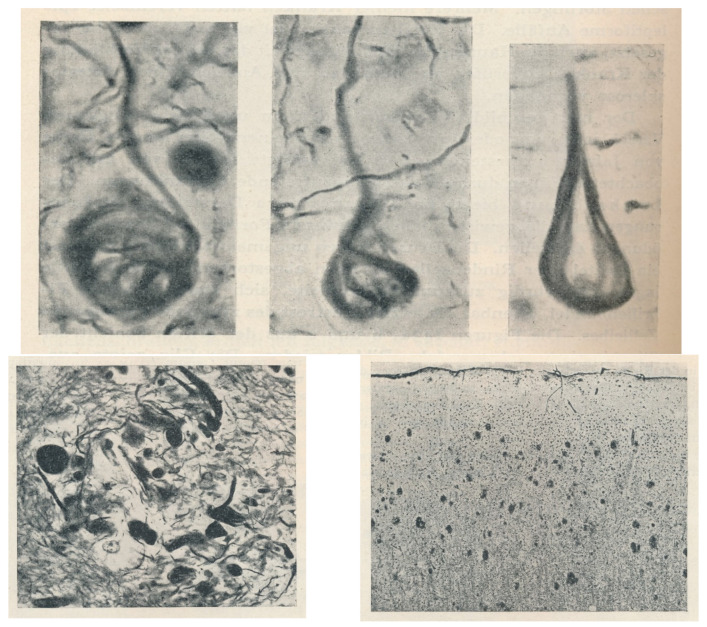
Pictures taken from de Kraepelin’ book [15]. NFT in the soma of cortical neurons (**top line**), a high magnification of NFT and neurites (**bottom left panel**), and NFT throughout the cortex (**bottom right panel**) of AD cases.

The history of AD began with the description of a new histological lesion in the brain of a female (Auguste D.), aged 56 when she passed away, who suffered presenile dementia (preSD; from Avicena to nowadays, the *senium* began at 60–65 years of age [3,4]) over a five-year period. The German psychiatrist Alois Alzheimer presented this case with the features of the later called *neurofibrillary tangles* (NFTs) in a meeting of psychiatrists in Tubingen in 1906, but his first publication was in 1907 [11] (without any figures). The histological new lesion was described by Alzheimer in the soma of cortical neurons: “*peculiar changes of the neurofibrils*”, “*one or more fibrils stand out*” (in the cells) … (had) *unusual thickness and the unusual ability to take up stain*” (Bielchowsky’s silver method), “*these are eventually seen clustering together*”. With the description of this case and three more cases by Bonifiglio and Perusini, medical investigators working with Alzheimer [12,13,14] in the Royal Psychiatric Clinic in Munich, the almighty Head of Psychiatry, Emil Kraepelin, director of the clinic and Alzheimer’s mentor, established a new disease. Kraepelin introduced AD in 1910 in his textbook of Clinical Psychiatry [15,16], the worldwide psychiatry rule textbook. Several histological images of the newly described lesions were presented in this textbook (Figure 1).

One year later, in 1911, Alois Alzheimer published a new extensive medical article [17] describing another AD case (Josef F.), with very different histological findings: his brain lacked NFT, exhibiting only SP. 

**Figure 2:**
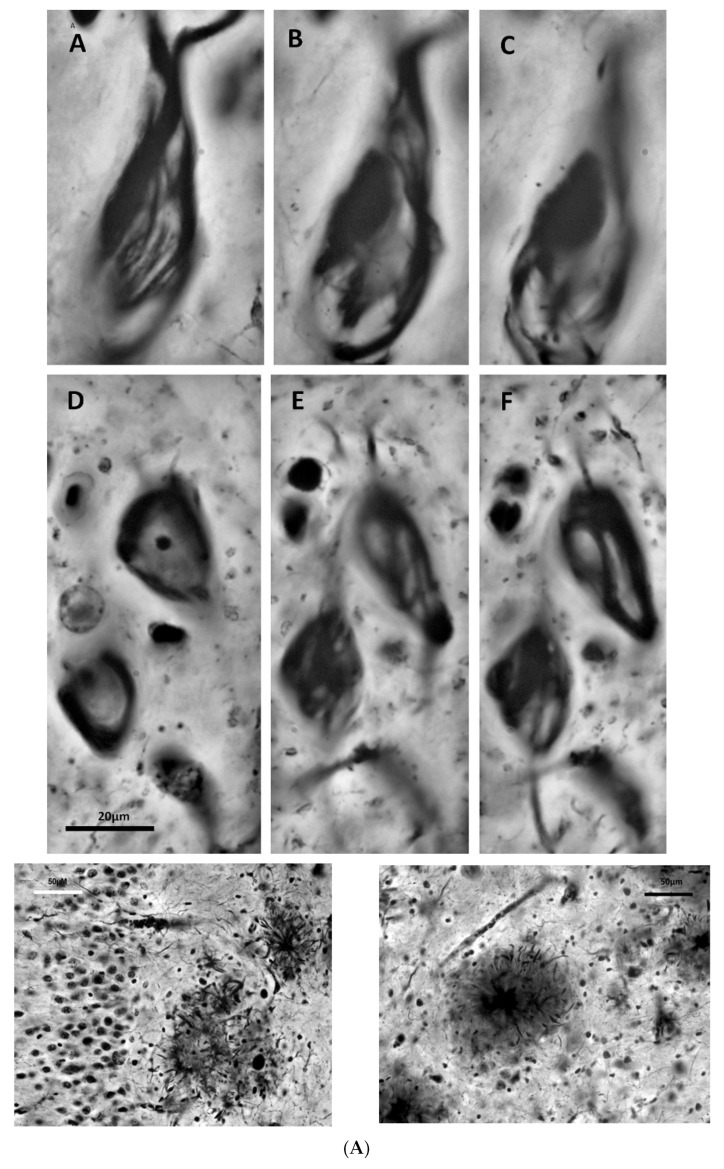

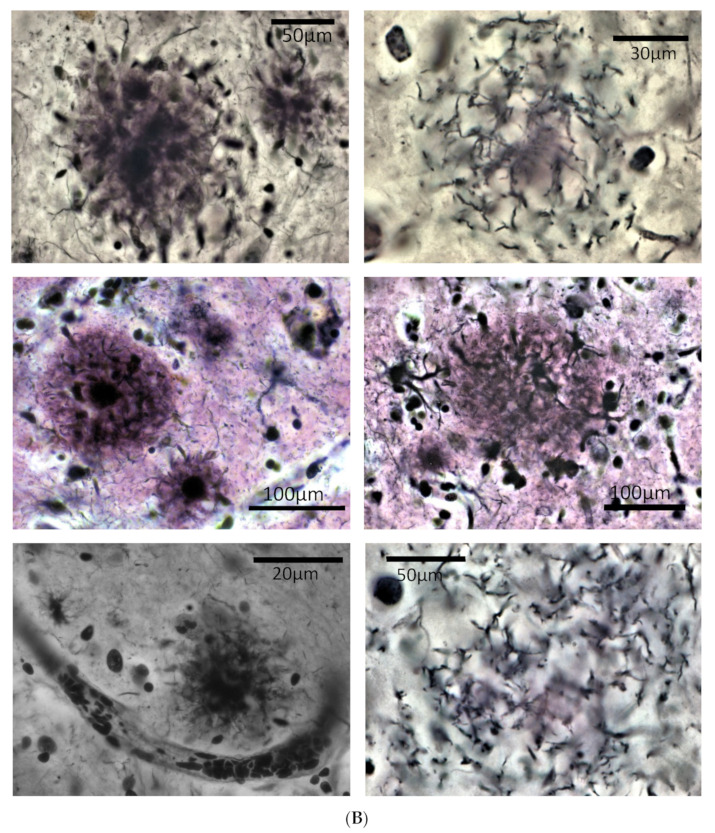
Cajal’s histological images of AD lesions [32]. (**A**) Neurofibrillary tangles (NFTs) (images (A–F)); neuritic plaques (bottom line). Bielschowsky staining. Courtesy of R. Martínez, Director of Cajal’s Institute in Madrid. (**B**). Details of neuritic plaques. Neuritic plaques stained with ammoniacal silver oxide (top line), formalin bromide (medium line, left) or sublimated gold chloride staining (medium line, right). Neuritic plaque near a capillary vessel (bottom line, left; reduced silver nitrate staining). Dystrophic neurites (bottom line, right; ammoniacal silver oxide staining). Courtesy of R. Martínez, Director of Cajal’s Institute in Madrid.

Oscar Fischer, a neuropathologist from the competing school of Prague (in the Austro-Hungarian Empire) led by Arnold Pick, published the most comprehensive histological work on SP in 1907 [18,19]. He described what were later called *neuritic plaques*, characterized by a central core surrounded by a corona of dystrophic neurites (Figure 2), and refined previous less accurate descriptions [8,9]. While Fischer considered SP as a hallmark of SD, he remained uncertain about their causal relationship.

Alois Alzheimer, in his 1911 article, used the eponym of Fischer’ SP (lost many years later [19]) and considered SP a normal histological finding of aging: “*the plaques are not the cause of SD but solely an accompanying feature of senile involution of the central nervous system*” [17]. He also included in this article the reasons why Perusini and Kraepelin separated PreSD from SD (each age has its own mental disorders, according to Kraepelin), although the pathological lesions in the brain were analogous [17]. In contrast, Fischer did not support this separation [19] and Alzheimer had doubts about it [12,17]. Both dementia types were recognized as a single nosological entity (AD) in 1978 [20]. (We list the most important contributors to this debate in alphabetical order [3,11,17,18,19,20,21,22,23,24,25,26,27,28,29,30,31,32,33,34,35,36,37,38,39,40,41,42,43,44,45,46,47,48,49], although the historian J Ballenger considers this vast array of commentaries mainly descriptive [50]).

**Figure 3:**
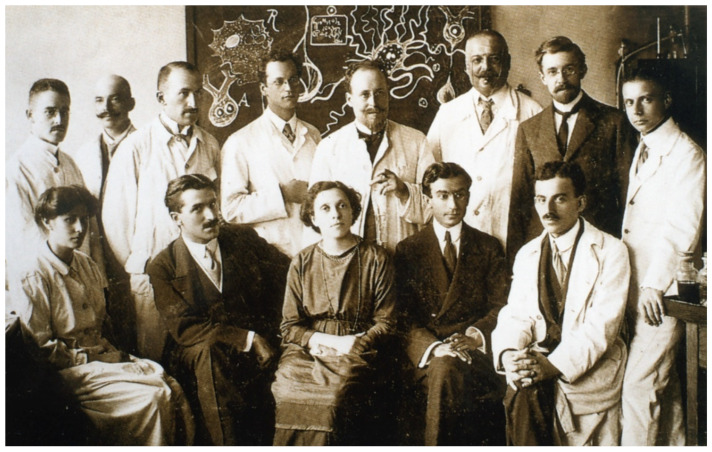
The Alois Alzheimer’s team. Alois Alzheimer and coworkers in Munich. Back (Left to right. Fifth: E. Kraepelin?; Sixth: A. Alzheimer; Seventh: N. Achúcarro, Spanish neurologist; Eighth: F. Lewy). Front (left to right): Frau Grombach; U. Cerletti; unknown; F. Bonfiglio; G. Perusini. Circa 1909–1910. Archive for History of Psychiatry, Department of Psychiatry, University of Munich. (Photography in sepia and permission to reprint by Psychiatrische Klinik für Psychiatrie und Psychotherapie in 2023).

Many of those responsible for the initial discoveries and discussions on AD and PreSD appear in an old portrait from the Royal Psychiatric Clinic in Munich (Figure 3). Berrios’ splendid historical article [12] explains: “*to identify the boundaries of the original description is no easy matter. The writings of Alzheimer, Fischer, Fuller, Lafora, Bonfiglio, Perusini, Ziveri, Kraepelin, and other protagonists in this story are deceptively fresh… In other words, a disease is defined not only by the power of resolution of the ongoing science, but also by periodic backroom decisions taken by her self-appointed mandarins*”.

Kraepelin established a new disease, presenile AD, with only four cases. Only twelve cases were described as such until 1912 but three of them were more than 60 years old [12,51,52,53]. Many of Kraepelin’s contemporaries and posterior pathologists in the first half of the 20th century [12,19,38,46,51,52,53,54] were not convinced that PreSD and SD were different diseases. Why did Kraepelin make the decision to establish this new disease? The explanation by Amaducci et al. [23] is very simple: the rivalry between Munich and Prague schools. This is a speculative but plausible explanation. Kraepelin wanted to enhance the prestige of his Munich team against the rival Prague team, and gave a premium (new eponym, Breslau Chair) to the serious work of Alois Alzheimer in the field of dementias. Beach [26] accepted this possibility and gave another argument: Kraepelin’s endorsement of Alois Alzheimer’s new disease could reaffirm his authority in the field of dementia and oppose Freud who considered Kraepelin as an enemy of his psychodynamic hypothesis about the genesis of dementia. In addition, Kraepelin an experienced clinician and taxonomist of mental disorders, had a firm belief in the influence of age in the genesis of mental illnesses (recall the dementia praecox, or schizophrenia) [3,25,55]. In fact, his decision established a new disease that endures today [12].

Kraepelin was wrong according to the historical evolution: the great majority of dementia series in the following 60 years showed that both presenile and senile AD has analogous histological characteristics (NFT and SP) with identical ultrastructure [37]. The separation of presenile and senile AD ended “officially” in a meeting in Bethesda in 1978 with 80 neuroscientists, who unanimously agreed that SD of Alzheimer type (SDAT) was identical to AD [20,37,56] and stated that there were no sound criteria to establish biological, clinical, or pathological differences. Kraepelin’s decision to separate PreSD from DS was denied, but Alzheimer won the eponym, and Oscar Fischer’s opinion was endorsed around 70 years later … such are the whims of history.

However, to better understand the early AD story, it is necessary to emphasize that the discussion and revision of the initial neuropathological descriptions were made more than 50 years after the first paper of Alois Alzheimer. Ironically, AD was a very rare disease almost completely unobserved by neuropathologists, and obviously by society, for more than a half century [24,25,26,42]. Before 1975, only 42 papers are registered in Medline under the keyword “Alzheimer” [27]. 

Fischer’s SP (now without his eponym) did not end as badly as his creator (who was tortured to death by Nazi police) [36], but the obliteration of the eponym did not prevent a profound discussion about this lesion, causing persistent and growing controversy [8,9,13,18,24,26,38,41,46,53]. Firstly, because in many studies, the correlation of SP with dementia or cognitive decline is weak [57,58,59,60,61,62,63] or almost non-existent [64]. Secondly, because from 1991, with the description of the amyloid cascade hypothesis (ACH) [65,66], SP was incorporated into the possible causal physiopathology of AD and considered a target for therapeutic drugs [67,68,69,70,71,72,73].

The period 1920–1970 is often described as a *dark time* for AD, marked by scarce data in the literature [41,74]. Ballenger noted that during this period “*American psychiatrists wrote extensively about age associated cognitive deterioration from a psychodynamic perspective*” [74]. To understand the later evolution of the dementia concept, it is useful to note several facts:

(a) In the first quarter of the 20th century, older people were often subjected to derogatory labels. William Osler, an eminent American physician, once quipped: “*a man over 60 years was useless and …jokingly, outline a proposal …, following a year comfortably ensconced at a college created for the purpose, a peaceful death by chloroform*” [24]; it is an anecdote, but very demonstrative.
(b) The second quarter of this century saw the psychodynamic Freudian model of dementia, the rise of social gerontology and the fight against senility [41,75]. This movement led initially by David Rothschild in America, with the influx of psychodynamic theories, posited that personal conditions, and not only SP (or other biological brain lesions), determine the manifestation of SD [41,43,75]. Rothschild’s histopathologic studies, along with others [74,75,76], showed a tenuous correlation between the presence of dementia and its presumed pathological determinants (many of the cognitively normal elderly had NFT and SP in their brains). These findings were confirmed in the following years [57,58,59,60,61,62,63,64] and fueled Rothschild’s opinions, and those of his followers, about the importance of personal circumstances (premorbid personality), disruption of family support, and social isolation in SD, away from the mechanistic biological causes represented by brain lesions. McMenemey comments: “the reserve power of the brain” explains the different individual capacities to cope with the organic brain lesions [43,67,77]. The concepts of cognitive and brain reserves (CRs) will reappear years later in dementia research [78,79,80,81,82] and other cerebral disorders [83,84].
(c) World War II catalyzed significant economic and social progress in the USA, shifting the focus from senility as a productivity issue to facilitating elderly leisure activities and fostering interest in “successful aging” [24,50,74,85].

In this new era AD, once a rare disease, was rediscovered as a strong paradigm [86] for professionals, society, and the media, marking the beginning of its splendid present as will be presented in the next epigraph. However, in this splendid new era German neuropsychiatry has been questioned and revised. Most charts and brain slides of the two first cases of AD were found after several years of intense search in the nineties, and a book [42] and several articles [38,87,88,89,90,91,92,93,94,95,96,97] revised the findings. Graeber et al. [89,92] insisted on the correct histological diagnosis of Alzheimer’s case 1. The possibility of a genetic cause (mainly a PS1 mutation) [93,94] was denied in a second study [95]. Although, a medical writer in the journal *Science* assured that the first case of AD was certified by reanalysis [91], he did not mention the Amaducci’s diagnosis of Auguste D. as metachromatic leukodystrophy [21,38]. The spinal cord of this case was not found …. The second case, Josef F., was extensively reevaluated with his family history since 1830 [96,97], indicating a familial disorder with an unknown dominant trait. The revision of the brain slides did not show NFT; this case could be currently considered as “AD plaques only” [22,98,99] because there were no Lewy bodies. But in the 21st century, this concept was eroded, considering that the “only plaques” cases always have some additional pathologies, or only appear in the oldest old [22,100,101,102]. The current AD pathology concept [103] eliminates these cases from AD pathological series [100] and are conceptually out of AD pathology [104,105]. 

In summary, currently SP (now without the Fischer eponym) are considered one of the two main pathological hallmarks of AD (not from aging, as Alzheimer and Fischer thought), PreSD and SD (SDAT) are a unique disease, and the two first cases described by Alois Alzheimer are not sound cases of AD according to the current AD concept. The historical discovery of AD has a controversial past from the 21st century perspective.

### 3.2. The Splendid Present of AD: A Medical Entity

#### 3.2.1. The Beginnings

The splendid present of AD began in the late 1960s and 1970s of the XX century when three circumstances opened a new era in the study of dementia and AD [24,25,36,41,49,50]:

(a) Socioeconomic development and health care produced an increase in life expectancy and old people became an important part of the global population. Myriads of people required care in asylums and paved the way to an important social movement [106].
(b) New technical capabilities such as the appearance of electron microscopy, and new histochemical, histological, and biochemical methods increased the possibilities of dementia study [33,34]. Two examples: Kidd in 1963 [107] described the ultrastructure of NFT (paired helical filaments) and Terry et al., in 1964, identified the amyloid core of neuritic plaques [108].
(c) A handful of neuroscientists began a reconceptualization of AD, the aforementioned *changing view* [25,26,56]. The psychodynamic approaches to dementia suggested some possibilities of improvement in the care of the elderly and social psychiatrists emphasized the importance of psychosocial factors as determinants of senility [25,41,74]. The historian Ballenger [24] wrote that this conceptual change was promoted in USA for two reasons: the fear of aging and the societal preference for its medicalization [109,110]; a disease such as AD was more acceptable than aging as a cause of dementia. Doctors, patients, politicians, and social media accepted this conceptual framework [36,41,106].

At the same time the pathological studies of the AD brain grew and were consolidated, especially in the United Kingdom. Early in the century, British science spoke of “*abiotrophy*” (i.e., premature cell degeneration) more than senility, and AD practically disappeared until 1950 [4,5,12,77,109]. In addition, the British neurologists and pathologists were not infused by psychoanalytic theories. In the sixties, pathologists such as JAN Corsellis [111,112] and Sir Martin Roth and his team (the Newcastle group) [113,114,115] studied long series of older brains from both normal and demented patients and applied quantitative methods to answer the following main questions: (1) the relationship between cerebral pathology (SP, NFT, vascular lesions) and the psychiatric diagnosis; (2) the relationship between intellectual deterioration and cerebral pathology; and (3) the relationship between normal senescence and these lesions. Their conclusions were the following: First, their quantification methods were reproducible by different scientists. Second, SP and NFT, as well as vascular lesions, were present in normal people without cognitive expression. Third, the quantitative measure of SP correlated with the severity of dementia in the majority of cases (although … “*In severely demented subjects and those diagnosed clinically as ‘senile dements’, the correlations between psychological and pathological measures decline sharply*”) [114]. Fourth, some cases did not exhibit correlation between the two measures. Later, Wilcock and Esiri [116], in a more detailed topographical study of cerebral cortex obtained better correlation between severity of dementia and pathological lesions in specific parts of the cortex, mainly NFT in the hippocampus and temporal cortex. In contrast, SP had a weak correlation with dementia. Mann [117] highlighted the echo of Roth and said: “*raise the level of awareness of, and interest in, the dementias of later life among clinicians and researchers alike*” … *dementia in later life were put on a ‘sliding scale’ of change, there being a threshold level of damage beyond which normal brain function was incompatible.* This pathological work was outstanding at that time.

#### 3.2.2. USA Scientific Establishment Certifies AD as the Main Cause of Dementia

This milestone was largely due to the herculean work of the three “*Bobs*”: a pathologist (Robert Terry) [118], a neurologist (Robert Katzman) [119], and a psychiatrist and gerontologist (Robert Butler) [120].

Terry and Katzman, who collaborated many times, produced extensive and determinant scientific work in the field of AD. Terry continued, with the aid of the electron microscope, the work of British investigators to determine the biological bases of AD [20,22,25,48,62,78,98,108,121,122,123,124,125]; Katzman [20,25,37,56,62,78,98,121,124,125,126,127,128,129,130,131,132] changed the AD concept with his clinical definitions, and his historical and epidemiological surveys. The social and political contributions of Robert N Butler, who coined the term “ageism” [133], a process of systematic discrimination against old people, promoted activism in the study and treatment of ageing [24,36,49,50]. He authored many scientific articles and several books on aging [133,134,135,136,137], was awarded the Pulitzer Prize for his collaboration in the mass media and was named the first Director of the National Institute on Aging (NIA) from 1975 to 1981 [24,36,49,50,106,138]. The three Bobs had a brilliant companion, Zaven Khachaturian, who oversaw the NIA extramural research program, created a network of Alzheimer’s Disease Research Centers and promoted AD from many perspectives [24,36,41,49,138,139,140,141,142,143,144]. He was the founding editor-in-chief of the influential journal *Alzheimer’s & Dementia* from its inception in 2005 until 2022.

Among the USA federal health research agencies created after the Second World War, the most important for dementia/AD was the NIA. It provided immense funds for health research, and had an important role in the field of neurological diseases. Nixon’s “War of Cancer” [138] was an example imitated by the NIA to obtain generous funding for the war against AD (the “most dreaded diagnosis”, “the loss of self”, “living dead disease”). In his recent book about AD [138], K Herrup describes the main purposes of the NIA: “*The first goal was to make AD more sinister”* (this was possible thanks to Katzman’s wonderful editorial about AD as a *major killer* [126]), and “*the second prong of the new approach was an energetic push to greatly expand the meaning of AD*”. The change in view of AD as a rare presenile disease to the new idea of a unified presenile and senile AD reached this prong [20,25,56]. The idea was intelligent and put into practice perfectly. The NIA’s objective to obtain a cure for AD (“the health politic of anguish”) [24] was put into action [36,49,74,106,138] and its result for the funding of AD research was incredible. The NIA’s budget for AD research soared from USD 19.3 million in 1976 to USD 222.6 in 1989 [106]. This figure is low in comparison to current NIA expenditure of 2.6 billion (10^9^), with about two thirds going to AD research [138]. 

Voluntary health organizations unified and launched the Alzheimer’s Disease and Related Disorders Association (ARDA) in 1979, now named Alzheimer’s Association (AA), with a lobby in Washington [24,36,49,74,106,136,137,138]. This association is recognized for becoming the second largest funding source for AD research in the USA. 

In this summary, it is not possible to describe the complex history of the social movement to establish the goal of defeating AD in the new North American society; it is well explained in a series of articles and books [24,25,36,41,49,56,74,106,136,137,138]. The NIA’s policy prioritized AD research while undervaluing the biology of aging research, but it determined critics in the medical establishment, such as those from Adelman (Director of the Gerontology Department of Michigan University) [145,146], followed by a controversy [147,148,149] extended over many years [36,41,49,147,148,149,150,151,152]. Another controversial research policy of NIA is clearly exposed in the article by Chaufan et al. [152] about the caregivers’ movement: “*The characterization of AD as a leading cause of death among the elderly was crucial to sustaining the movement, but also operated as a “double edged sword”. By construing “senility” as a treatable, even curable biomedical entity, operated as a “double edged sword” … compounding the effect of conservative federal policies that emphasized the treatment or cure of AD, and less so the long-term care service needs of sufferers and caregivers that had given rise to the movement*”. This antithetic problem and the corresponding discussion continue in the 21st century [36,41,138,149,150,151,152,153,154,155] because the American administration was unable to fund many aspects of care of AD patients and their families [36].

#### 3.2.3. The Internationalization of AD

**Figure 4:**
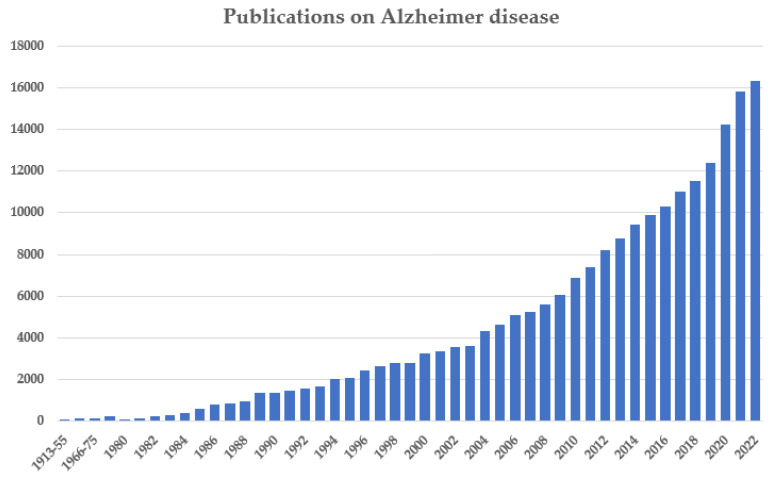
Publications on AD recorded in Medline by year: 1970–2022. (Taken from Medline in 2023).

Gradually, beginning in the 1970s, but intensifying in the 1980s and 1990s, the American concept of AD and its accompanying social and institutional movement became international. Figure 4 illustrates the quasi-exponential growth of AD research as reflected in Medline publications. This graphic reveals a limited number of articles in the 1970s, with a clear increase in the 1980s and 1990s, exceeding 3000 publications per year in the 21st century, increasing to more than 14,000 per year in 2020 onwards. Detailed analyses of the publications, patents, clinical trials, funded research, and so on, by time periods, countries, and continents [156,157] show that the rank of publications in the field of AD period from 1983 to 1997 was 1st USA and 2nd UK; but from 1998 to 2002, the rank was 1st USA, 2nd Japan, 3rd UK, 4th Germany, and 5th France. From 2008 to 2017, the rank changed: 1st USA, 2nd UK, 3rd China, 4th Germany, 5th Italy, 6th Japan, 7th France, 8th Canada, 9th Spain, 10th Australia, and 58 countries publishing more than 11 papers/year. In fact, AD is an international disorder with more than 50,000 papers, written in 157 countries during the period from 2013 to 2017 [157].

The modern history of AD as a global disease can be presented, as Bondi et al. performed, by periods in years [28]. However, we prefer to present the data around the following points of interest: (1) some important milestones in the history of AD (see Table 1 and Table 2); (2) evolution of the definition of AD; (3) the most important hypothesis of the causation of AD; and finally (4) the main XXI century novelties of this disease.

#### 3.2.4. Important Milestones in Evolution of AD

Table 1 presents key milestones in the evolution of AD research, highlighting the primary authors of these developments. This selection, though subjective, is based on the first or distinctive description of these innovations, published in the most relevant journals or ranked highly in Medline *best match* [10], and supported by large numbers of Medline references.

In Table 2, we present the main pharmacologic treatments with commercial use employed in SD (of unknown etiology) and/or AD, which have historical interest. It is only a summary of the most representative publication of each drug treatment selected from more than 40,000 Medline references about the pharmacologic therapy of dementia/AD. Selecting these examples was challenging. For instance, Hydergine is cited in over 1000 Medline references and was approved by the FDA for use against dementia in the seventies due to its effects recorded with the scale Sandoz Clinical Geriatric Assessment in controlled trials, but it was abandoned in the eighties due to the uncertain clinical efficacy [188]. Another example would be Nimodipine, which has more than 4700 Medline references and the approval by the FDA (1988) was only for managing vasospasm following subarachnoid hemorrhage, but its use in dementia started in the eighties due to a possible beneficial vascular effect [189] tested in several trials of vascular and non-vascular dementias. The drug did not demonstrate clear efficacy in dementia, although its effects in vascular cognitive impairment [190] and subcortical vascular dementia [191] were better than those of placebo. Current and forthcoming treatments are also listed in Table 2 and will be briefly discussed in subsequent sections.

**Table 2:** Brief history of pharmacological treatment of SD/Alzheimer disease.

Year/Author	Drug Treatment
1984/Hollister and Yesavage [192]	Hydergine (vasodilator, metabolic enhancer) *
1986/Growdon et al. [193]	Piracetam (nootropic): unproven efficacy in AD
1986/Summers et al. [163]	Tacrine (AChEI) the first drug FDA approved for AD
1989/Thal et al. [194]	Vinpocetine (vasodilator, enhancer): unproven efficacy
1995/Saletu et al. [195]	Nicergoline (vasodilator, enhancer): unproven efficacy
1995/Fritze and Walden [189]	Nimodipine (calcium antagonist) unproven efficacy in AD *
1996/Rogers et al. [169]	Donepezil (AChEI) for AD, the first AChEI widely prescribed
1999/Rösler et al. [196]	Rivastigmine (AChEI)
1999/Winblad et al. [197]	Memantine (NMDA antagonist)
2000/Raskind/Tariot [198,199]	Galantamine (AChEI)
2002/Orgogozo et al. [172]	Elan AN-1792, active anti-amyloid vaccine in humans, is halted
2005/Schneider et al. [200]	Ginkgo biloba is not effective to treat AD
2008/DeKosky et al. [201]	Ginkgo biloba does not prevent AD
2014/Salloway et al. [202]	Bapineuzumab (AAbmA) phase III trials failed in AD
2014/Doody et al. [179]	Solaneuzumab (AAbmA) phase III trials failed in AD
2018/Rosenberg et al. [203]	Multidomain intervention reduces cognitive decline (FINGER)
2022/Haeberlein et al. [204]	Doubtful efficacy of Aducanumab, but FDA approved
2023/van Dyck et al. [205]	Small efficacy of Lecanemab in early AD
2023/Sims et al. [206]	Small efficacy of Donanemab in early AD

AChEI—Acetylcholinesterase inhibitor; AAbmA—anti-amyloid-beta monoclonal antibody. * See text.

#### 3.2.5. Evolution of the Concept and Definition of AD

While a precise definition is essential for managing and researching a disease, the definition of AD has evolved significantly over time (see Table 3). 

Until 1984, the AD definition was pathological, including SD in the seventies, with the creation of SDAT by Katzman [20,25,56]. McKhan [162] introduced a “modern” definition of AD in 1984 (co-work of NINCDS and ADRDA). This definition was clinico-pathological and clinically probabilistic (categorizing AD as possible, probable, and definite), using pathological examination as the confirmatory criterion. This definition was very influential in clinical practice. Several pathological validations of its diagnostic criteria were undertaken in two long series [207,208] with many cases and in several other series with more than 50 cases [209,210,211,212,213,214,215,216,217]. In general, these clinical criteria have good accuracy and sensitivity (~90% in probable AD, and ~80% in possible AD), but moderate-poor specificity (70–40%) [207,208,209,210,211,212,213,214,215,216,217]. AD associated with Lewy Body dementia (LBD) was not excluded in most of these series.

The separation of AD with dominant familial inheritance began in the mid-eighties, when it was demonstrated that the mutations in three known genes (APP, PS1, and PS2) caused the majority of early familial autosomal dominant AD (ADAD) [175]. The ADAD, purely genetic, represents only 1% of cases, whereas the sporadic, late onset, (SAD) represents 99% of the AD cases [161,175,218]. 

**Figure 5:**
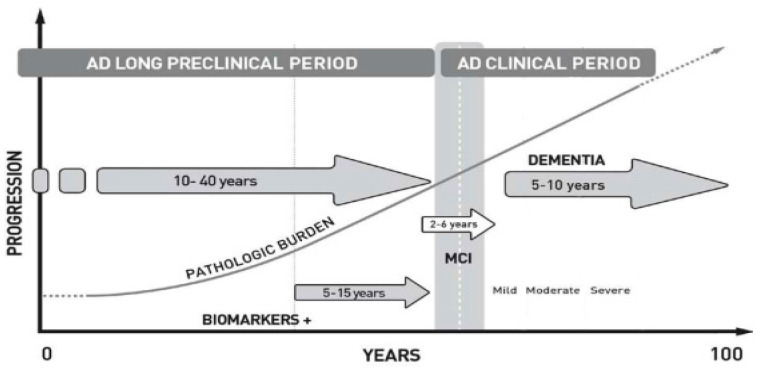
Putative AD evolution. Possible AD brain and clinical evolution along life. Theoretic evolution of AD cases with increasing pathologic burden along life, going from the early preclinical period to preclinical period plus positive biomarkers (+), predementia (MCI), and progressive stages of dementia. (The time-periods described are an average). Many cases with great AD pathologic burden never exhibit MCI or dementia and die with normal cognition. Pathological burden in the oldest cases is always mixed. Modified from Bermejo-Pareja F [224]; and inspired in Scinto et al. [225].

By the end of the nineties, two different approaches to AD research determined a new development and expansion of the concept of AD. The first came from pathological studies by two German pathologists (Braak and Braak, and their team). They evaluated a large series of 2366 brains of different ages from Frankfurt University and described the evolution of the main pathological AD hallmarks across the age span. This systematic and extensive study emphasized the long-lasting preclinical appearance of AD pathology and established six stages in its overall evolution based on the NFT distribution and quantity [168,219,220,221,222,223]. The pathological hallmark of AD can be observed in the brain as early as childhood and increases throughout adulthood and old age of the future AD patient [224,225] (see Figure 5). 

The other relevant approach came from the increasing clinical interest in the complaints and evidence of age-associated memory and cognitive deficits that were described using several operative definitions and criteria [158,224,225,226,227,228]. “Mild cognitive impairment” (MCI) [170], became the most widely recognized condition, and it was internationally accepted, firstly for research, but later as a clinical entity that has prompted an immense literature (nearly 90,000 Medline citations), mainly because early AD is probably its main cause [229,230,231,232,233,234]. The MCI identification and diagnosis were considered a good opportunity to prevent dementia and especially AD [229,230,231,232,233,234]. Khachaturian [235], in his authoritative state-of-the art of the AD diagnosis published in 2005 [235], indicated the MCI was the way to prevent AD from its prodromal state. 

These historical developments have broadened the nosological concept of AD: the predementia stage was incorporated and the study of AD biomarkers (in CSF, blood, and neuroimaging) became crucial [214,236,237,238,239,240] to support the diagnosis of AD mainly in this initial stage. Subsequently, in 2004, the well-known project “Alzheimer’s Disease Neuroimaging Initiative” (ADNI) was launched [36,41] and one of their main goals was the investigation of the clinical features and biomarkers of the early stages of AD that could be treated in drug trials. 

In 2007, renowned French neurologist Bruno Dubois and colleagues [241] introduced new research criteria for AD, as an extended process characterized by clinical and biomarker features, and later the prestigious NINCDS-ADRDA criteria were also updated including biomarkers [242]. In 2013, the DSM-V (the manual of classification of psychiatric diseases) [243] proposed replacing the term MCI by “minor neurocognitive disorder” (and “major neurocognitive disorder” instead of dementia), but both propositions had scarce clinical success. MCI and “minor neurocognitive disorder” have analogous characteristics: they are clinically fuzzy, have multiple etiologies, and convey a 2 to 9 fold increased risk of dementia in their evolution [244], although the great majority of MCI patients (in population-based series) do not evolve to dementia and some of them revert to normal cognition. Figure 5 represents a theoretical evolution of clinical features, brain pathologic burden, and biomarkers of AD cases.

In the past decade, both the NIA-AA guidelines and Bruno Dubois’ group have been pioneering a new biomarker-based concept of AD with research definitions for preclinical [245], prodromal and clinical stages [245,246,247,248,249]. The final path in this direction has been the definition of AD by Jack et al., in 2018, as a purely biological entity without the historical clinical features of cognitive decline and dementia [250]. The new biologically defined AD is much more prevalent than clinically defined AD due to asymptomatic individuals with subclinical pathological burden [251]. This new AD definition as a research framework is supported only by biological biomarkers classified into three categories: (A) β-amyloid deposition; (T) pathologic tau; and (N) neurodegeneration. The AT(N) system is designed for classifying cases according to their biomarkers in blood, CSF and in neuroimaging (mainly PET and MRI), not according to pathologic data or formal clinical diagnosis of dementia [250,251]. The elimination of the historical Terry–Katzman–McKahnn 1984 clinicopathologic concept has generated many criticisms. It is well known that the AD pathologic diagnosis is probabilistic (without a gold standard) [252], but it provides more precision in the classification of cases than the postulated biomarker models. Many current pathologists [105,253,254,255,256,257] do not support the reductionism of the new research framework of AD “pathology”. Firstly, because it lacks known pathological validation and its clinical published validation was negative [258]. Secondly, because the biomarker criteria suggested are only probabilistic with many limitations expressed by several authors from all over the world, including China [259,260,261,262,263,264,265]. The data from the ADNI project results indicated that only summing up biomarkers to clinical and psychological data (the most robust markers of AD) ameliorated the probability of a better prognostic significance [266,267]. The NIA-AA proposal is flexible and accepts that new biomarkers and biomarker categories can be added in the future. It also emphasizes its intended use for observational and interventional research, not for routine clinical care, and its usefulness to test alternative physiopathological hypotheses about the interactions among different pathologic processes (denoted by biomarkers) and cognitive symptoms. Obviously, this proposal facilitated the AD trials and has been accepted by many investigators. Otherwise, numerous critiques have emerged regarding the new biological criteria for AD [268,269], some of them being severe because they affected the methodology and its ethical aspects [138,269,270,271,272,273]. Therefore, in this very critical context of biological and clinical breakaway, the relevant modification of the new conceptualization of AD presented by Dubois et al., in 2021 [274], with the inclusion of clinical dementia in its definition is comprehensible. 

**Table 3:** AD diagnostic evolution (selected data).

AD Diagnostic Evolution	Year/Author
*Clinico-pathological diagnosis*	
AD (presenile dementia) vs. senile dementia	1910 Kraepelin [15]
Presenile AD and Senile Dementia of Alzheimer Type	1978 Katzman [20]
AD-Clinico-pathological disorder	1984 McKhann [162] *
Pathologic validation of McKhann criteria [162]	Several authors [207,208,209,210,211,212,213,214,215,216,217]
Familial-genetic (early AD) and sporadic AD	1985 Glenner [161]
Mild cognitive impairment	1999 Petersen [170] **
*Biological diagnosis*	
AD Research criteria	2007 Dubois [241] ***
Clinico-pathological disorder plus biomarkers	2011 McKhann [242]
Biological definition of preclinical AD	2011 Sperling- NIA-AA [245] $*
Revision of the AD Dubois [241] definition	2014 Dubois-IWG-2 [248] $*
AD with and without dementia (continuum)	2016 Dubois [249] $*
AD definition as a biological construct	2018 Jack-NIA-AA [250] $*
AD clinical and biological	2021 Dubois-IWG [274] $ *

Main medical criteria: * NINCDS–ADRDA criteria: Probabilistic diagnosis; ** Diagnostic differentiation from AD; *** Clinical validation negative [258]; $*: No validation. DSM (Diagnostic Statistic Manual) [243] diagnostic criteria not commented.

#### 3.2.6. AD Hypotheses

There are many AD causal hypotheses [275,276,277,278]. The most important are presented in Table 4, with the main references of the corresponding author [48,62,65,66,140,160,168,187,279,280,281,282,283,284,285,286,287,288,289,290,291,292,293,294,295,296,297,298,299,300,301,302,303,304,305,306,307,308,309,310,311,312,313,314,315,316,317,318,319,320,321,322,323] and according to their bibliographic load. For the selection we reviewed Google Scholar (the widest selection), and to obtain a literature quantification we selected the number of Medline references (more than 9000).

Several points should be emphasized. First, the hypotheses about AD etiology cover a wide range, from undernutrition to multiple causality and from synaptic function to neural membrane aging. Second, several causal hypotheses have undergone multiple modifications over time and many of them are some mixture of two or more colligated (e.g., normal-pressure hydrocephalus, and senescent changes in CSF, or oxidative stress and inflammation [324]). Third, many hypotheses are not mutually exclusive, but rather complementary. Fourth, the high failure rate (99.6%) of AD therapies [325] has spurred the development of new hypotheses. Finally, some hypotheses are unconventional, like the idea of AD being akin to cerebral glaucoma … [326]. In this review, we can only comment on the most representative hypothesis: the amyloid cascade hypothesis (ACH). The length of this article prevents us from commenting on other hypotheses, such as aging, the second hypothesis, that is too complex to be discussed.

The ACH is the most referenced AD hypothesis and the most influential from a biological and therapeutically point of view (see Table 4). Two geneticists, Hardy and Higgins, edited this hypothesis in 1992 [66]. Their main argument was: “*Deposition of the amyloid β protein… is the causative Alzheimer’s pathology*”. According to this article, the other hallmarks of AD: NFT, cell loss, vascular damage, and dementia, are consequences of the brain Aβ peptide deposition. The most salient support of ACH is the abnormal cleavage of APP protein and the increased production of Aβ in the ADAD due to mutations in APP, PS1, or PS2, or in Down syndrome due to the three alleles of APP [164,165]. This hypothesis has been tested in several transgenic mice, as well as the activity of many drugs against AD, but these models do not replicate the AD human pathology, they only show an excessive amount of Aß [138]. In a subsequent paper in 2002 [279], Hardy and his co-worker, Dennis Selkoe, whose laboratory studied cultured cells and enabled the identification of Aß production and some potential inhibitors, presented again the hypothesis that the excess of Aß is cytotoxic for neural tissue [327]. Their paper [279] clearly formulated the ACH, implicating Aß oligomers in the toxicity (neuritic injury, synaptic loss), inflammation, and oxidative stress [138,279]. Both delineated the potential bases for AD therapy [279]: “*by the use of active or passive Aß immunization, in which antibodies to Aß decrease cerebral levels of the peptide by promoting microglial clearance and/or by redistributing the peptide from the brain to the systemic circulation. Although active immunization with synthetic Aß1-42 peptide produces robust benefits in APP transgenic mice without detectable toxicity*”. Despite its dominant position, the ACH has also had negative comments [49,138,328,329], cases difficult to explain by the hypothesis [330], and modifications such as a ‘bioflocculant hypothesis’, which posits that Aß is normally produced to bind neurotoxic solutes (such as metal ions) [331]. Joseph et al. [332] stated this demolishing critic: “*Just as the Church at the end of the Middle Ages was comfortable with the idea that the earth was the center of the universe, the Church of the Holy Amyloid (CHA), at the beginning of the 21st century, is comfortable with a similar role for amyloid beta in the Alzheimer universe*”. These authors and other followers [333] consider that the defenders of the ACH acted with religious blinders ignoring several important contradicting facts. The ACH has not sufficiently proven the brain neurotoxicity of Aß nor its role in the genesis of neurodegeneration and cognitive deficits of AD. It is good to remember that the vaccine against amyloid to treat AD was halted for adverse events in the same year (2002) [172] of the last ACH publication [279].

Probably the most serious theoretical challenge to this hypothesis was the precise description by Braak and co-workers regarding tau brain deposition throughout the life of many subjects showing that the NFT and ‘pretangles’ (the initial NFT) began many years prior to the appearance of SP (with its Aß central core) [168,219,220,221,222,223,285]. However, the ACH goes ahead and supports that “…*clinical trials need to be organized in the very earliest stages of the disease*” [334] with the expectancy of more clinical efficacy. This trend has been followed by many clinical trials in the last decade.

The most empirical challenge to the ACH has been the repeated failure of clinical trials with anti-amyloid drugs. After the failure of the active immunization (vaccine) against the Aß deposition in AD [172], the anti-amyloid-beta monoclonal antibodies (AAbmA) were developed for passive immunization [335,336,337,338,339]. In fact, bapineuzumab, a humanized AAbmA initiated its course as AD treatment in 2003 [340], and other similar AAbmA [341,342,343], as well as many other anti-amyloid drugs [73] followed this path. The trials with AAbmA in SAD [344] or in ADAD [345] have shown repeated negative results. Systematic reviews and meta-analyses of trials with these drugs consistently demonstrate a significant reduction or elimination of the brain amyloid burden, but no clear cognitive or behavioral improvement nor reduction in the progressive decline [346,347,348,349,350]. The Bayesian meta-analysis supported the null hypothesis [186]. These negative results of AAbmA, even after a long follow-up [351,352], have been also observed with many other anti-amyloid drugs [73,138]. On the other hand, the passive immunization determines adverse brain effects such as ARIAs (amyloid-related imaging abnormalities), whose prognostic significance is not yet well known [349,353].

These results generated the disapproval of the ACH of AD for many authors [73,138,354,355,356,357,358,359,360,361,362]. Some of them argue that the supporters of this hypothesis do not accept that it is *falsifiable* in the Popperian sense [363] and always attribute its failure to some methodological drawback (inadequate dosage, inappropriate treatment time, poor selection of patients …), but not to hypothesis falseness. As Espay et al. indicate [73], this is more a religious dogma than a scientific position [138,332,361]; the trueness of the amyloid hypothesis must be demonstrated in trials… [364]. D Smith (Oxford Emeritus Professor) [360] radically raises an ethical question: “*is it justifiable to ask patients to undergo yet more trials of anti-amyloid treatments?*” Other authors indicated the biological implausibility of the hypothesis for the cerebral protective role of amyloid burden [365,366] or other reasons [355,359,362,367]. In contrast, the creators of ACH and many followers sustained the vigor of this hypothesis [368] with new modifications [369,370]. The recent doubtful results of aducanumab [204], and limited positive data of lecanemab [205] and donanemab [206] in their phase III clinical trials have reinvigorated the supporters of ACH and provided good news for patients, families, and clinicians after more than 20 years of failures. Lecanemab has been approved by the FDA and the approval of donanemab is also expected, but they still have to demonstrate their therapeutic effect in real life, the number of patients who can benefit, and to what extent they modify the course of the disease.

The discussion of the role of amyloid in the pathogenesis of AD is too complex for this review [359,361,362]. The lack of or small effects of anti-amyloid drugs in clinical trials and the high prevalence of Aß deposits in the brain of aged persons with normal cognition (around one third at 75 years of age and 70% at 80 years of age) [371] seem to indicate that they are neither the first cause of AD nor the main determinant. 

**Figure 6:**
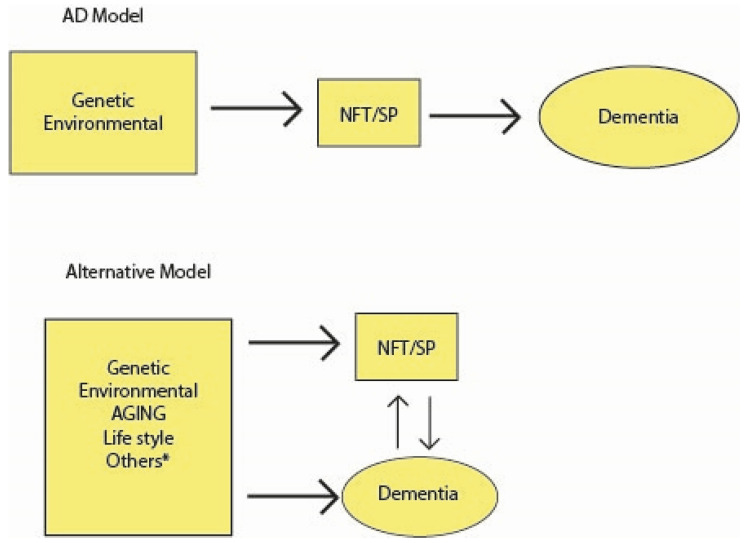
Possible Physiopathology of AD. This figure represents the AD physiopathological model of the cascade hypothesis (top) and a more complex relationship between many risk factors, dementia, and the hallmark pathologies of AD: Neurofibrillary tangles (NFTs) and senile plaques (SPs). Figure inspired in Herrup [138] and modified by authors. * There are many RF described for dementia/AD.

Figure 6 presents in a simple diagram the lineal pathophysiology proposed by ACH (top). Probably AD and other neurodegenerative diseases have a more complex set of causes, determinants, and drivers that interact with frequently mixed pathologies and events along the life (bottom). In Figure 5, we put the word “aging” in capital letters, because it is the main driver of many physiological derangements in these elderly conditions. The entanglement between aging and dementia/AD cannot be extensively discussed in this paper (see Section 3.3), but we underline that it is the second causal hypothesis according to the number of references on this subject in the literature [4,30,36,40,41,54,67,73,75,77,105,106,110,124,127,129,131,138,145,146,149,187,256,280,281,282]. 

#### 3.2.7. The 21st Century Novelties

Two main novelties emerged in this century: firstly, promising epidemiological findings; and secondly, challenging news for the established concept of AD: in the oldest old, AD pathology is not preponderant. These novelties change the conceptual framework of AD.

##### Dementia/AD Could Be Prevented, Epidemiological Discovery

The review of Haan and Wallace [173], at the beginning of the new century, posed a relevant question: is it possible to prevent dementia? They had a quick positive answer in the following years. Table 5 [182,372,373,374,375,376,377,378,379,380,381,382,383,384,385,386,387] presents the main studies that demonstrate that this question was opportune. In summary, most of the recent elderly cohorts followed up in well-developed countries show a decrease in dementia and AD incidence and better cognitive performance [372,373,374,375,376,377,378,379,380,381,382,383,384,385,386,387,388,389,390,391,392] in comparison with the previous cohorts. Japan stands out as an exception among affluent nations [388,393]. Prevalence of dementia/AD decline is in a steady state, or increasing because prevalence depends very much on life extension (improved in rich countries), and on the number of people at risk (increasing number of elders in affluent nations), and not only on the incidence of dementia. Obviously, the data on dementia and AD prevention do not possess the same level of certainty as clinical trial results; but epidemiology is quite a stable science, and its results are increasingly respectable. Notwithstanding, we should underline that Table 5 includes mainly cohorts in which the diagnosis of dementia and its subtypes (AD, or vascular dementia–VaD-) is clinical (usually without hospital-based biomarkers), and there is great variability in the methods, diagnostic criteria, time observation periods (in general greater than 10 years), and participant numbers (large cohorts) of the exhibited investigations.

The key takeaway of this good news is that the risk of dementia/AD is decreasing without any AD etiologic therapy. Similar findings occurred in the past with other chronic illnesses, e.g., tuberculosis or myocardial infarction [394,395]. Presumably, better life conditions, lifestyle, and a decrease in the environmental risk factors (RFs) in the new cohorts are the putative cause. 

The literature on the important subject of RF and prevention factors (PFs) for dementia/AD is immense (more than 28,000 references in Medline; more than 500 studies and reviews). We have selected only the main RFs in the summarized Table 6 (performed with reviews listed in alphabetical order) [185,396,397,398,399,400,401,402,403,404,405,406,407,408,409,410,411,412,413,414,415,416] and graded the RF authentication according to the number and quality of published studies and to the recommendations of several authors [399,416]. From an epidemiological point of view, it is impossible to recruit a large cohort of young people and track them until old age with many subgroups to distinguish the most determinant RF and prevention factors (PF) [417]. Clinical trials to assess life course and lifestyle are also difficult to conduct. 

Table 6 presents the main RF related to the incidence of dementia/AD and the evidence collected about each one according to the literature [185,396,397,398,399,400,401,402,403,404,405,406,407,408,409,410,411,412,413,414,415,416]. Probably 30 to 40% (one author elevated this figure to 66% [416]) of elderly progressive dementia/AD would be prevented (or postponed) by the clinical control of these RF [418,419,420,421,422,423,424,425,426]. Obviously, genetic factors cannot be controlled at this moment and are not presented in the table. There are many hot debates about the eventual circularity of some RF, such as depression or hearing defects, that could be (in part) consequence of the neurodegenerative process, and about the effects of their treatment. 

The number of RF/PF of dementia is very high; more than 90 have been described [416]. Many of them are not independent but strongly associated, as many reviews indicate [422], and act synergistically on the risk for dementia in a dose-response way. A recent meta-analysis estimated the risk according to the number of RF; one RF: 1.2 (95% CI 1.04–1.39), two RF: 1.65 (95% CI 1.40–1.94), three or more RF: 2.21 (95% CI 1.78–2.73) [422].

Dementia prevention has two main approaches and challenges: the individualized control of the RF in subjects at risk, and the public health policies to control these RF in the population. Several multidomain intervention trials for the control of multiple RF/PF have been undertaken and already tested [426,427,428]. The FINGER trial has given the most outstanding positive results [426] and it is currently replicated in several countries [429]. Measures to reduce smoking or air pollution are a good example of public actions. Many authors support these population-level approaches [430,431,432,433,434], including a recent recommendation by the WHO [184] that considers the necessity of public campaigns creating awareness of the main RF of dementia and AD.

##### Novelties about the Pathology of Dementia in the Oldest Old

We should remember that the birth of AD was a pathological discovery [11] and the references to this disease were preferentially pathological for more than half a century [11,18,19,20,42,57,75,76,77,111,112,113,114,115,116,117]. The splendid present of AD changed this situation [20,25,56,121,126,156,157]. The “*Nun study*” inaugurated a new era in the knowledge of pathology of dementia in the late nineties. This exceptional survey revealed one relevant finding: the cerebral vascular lesions play a significant role in the manifestation of dementia in persons with AD neuropathology. Another fact, known since the thirties, was noted: some *Nun study* sisters, even centenarians, had normal cognition with high AD pathologic burden [435,436].

**Figure 7:**
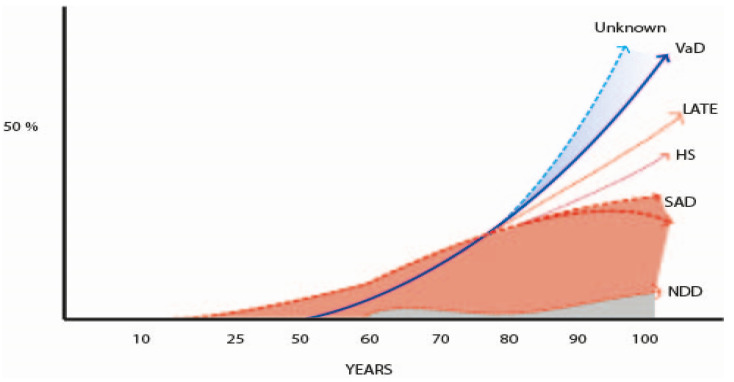
Pathological burden in the brains of patients with dementia throughout aging. This figure charts the brain pathologies in demented cases according to different periods of aging. Vascular lesions (VaDs) grow almost exponentially from the age of 50. The pathological burden of type AD lesions (SADs) could begin in childhood and possibly decline towards 90–100 years of age according to some studies [455] or increase (grey). LATE and hippocampal sclerosis (HS) increase with aging. Many other neurodegenerative disorders (NDD, e.g., ADAD, LBD) have an early presentation or increase with aging. Modified from Bermejo-Pareja et al. [387] and inspired by Nelson et al. [455] and modified, with data from other authors ([456,457,458,459,460], see text).

In the 21st century, with the increasing survival of the populations, several studies underlined that the neuropathology of AD, especially in patients older than 80–85 years of age (oldest old), is much more complex than it was believed in the previous century. This discussion was initiated by the findings in a Cambridge study in 2000, showing a marked overlap of different pathologies in demented and non-demented individuals [437] and was followed by the description of the TPD43 neuropathology at the turn of this century [438]. In fact, in the new century, several new pathologies are quite well established in the field of dementia [439], mainly the limbic-predominant age-related TDP-43 encephalopathy (LATE), whose prevalence rose to 40% in demented persons [440,441], but other less frequent new pathologies were also delineated [105,256,257,442,443,444,445,446,447,448]. But the most notable discovery in the pathology of dementia was that the mixed pathology (NFT, SP, plus vascular and other neurodegenerative disorders, such as Lewy bodies, argyrophilic grains, and hippocampal sclerosis) was the most frequent finding in the brain of demented persons [105,174,437,442,443,444,445,446,447,448,449]. These facts changed the previous concept of the AD pathology as the most frequent pathology in elderly dementia, that could be true in young-aged ADAD, but not in the oldest old. Another significant point is the large number of individuals without dementia that have high pathology burden of different types. This is an old well-known phenomenon [43,44,75,76,77,78,79,80] attributed to CR [80,81,82,83,84], but two cohorts of different genetic backgrounds demonstrated a similar percentage of CR [450]; genetic resilience? Another challenge is the observation that in around 20% of old patients with dementia its cause cannot be ascertained by pathological exam [178,451]. Could it be caused by the frequent vascular pathology associated with aging [452], or by still unknown proteomic deposits and synaptic dysfunction [257,453,454]? This situation points out the necessity of new approaches to the pathological study of dementia [257,439]. These issues are out of the scope of this clinical review. Figure 7 charts out the data from many authors that describe the main pathologies observed in the brain of old people according to their age [454,455,456,457,458,459,460,461,462,463,464,465]. Table 7 summarizes the main studies of the brain pathology in the oldest old demented people [456,457,458,459,460,461,462].

### 3.3. The Unpredictable Future

Compared to the large number of articles focusing on biomarkers (over 1500 in Medline) or other specific aspects of AD, those analyzing the future of dementia/AD as a general subject are rather limited [28,463,464,465,466]. Its most frequent topics are AD concept and definition, RF and PF, AD physiology and therapeutic targets, AD resilience/CR, and care and social aspects [467].

The most complex and difficult to guess is the future concept and nosology of AD, because currently it is not clear. Moreover, Richard and Brayne posited more than a decade ago that AD is not a disease but a syndrome: “*In its most common late onset form, the term Alzheimer’s disease is unlikely to refer to a discreet neuropathological entity, but to a diffuse clinical syndrome that represents the gradual accumulation of multiple pathologies, which arise from risk factors over the course of life*” [468]. 

The current medical knowledge has progressively shown that SAD is a very heterogeneous disorder. From the genetic point of view, it is polygenic. More than 70 genetic loci, together with ApoE, determine the risk of AD [175,218,469,470], with different actions according to the ancestral origin [471]. In addition, epigenetic variables, and microRNA, associated with environmental factors, complicate the biological endeavor and the phenotypic manifestations of AD [472,473,474]. Our genetic understanding, including GWAS, is still quite limited due to missing hereditability, and difficult to improve [475,476]. 

The same landscape is for the clinical manifestations. The most frequent is a progressive memory deficit (45–85%), initiated as MCI [244,477,478], but atypical presentations (visuospatial, language, executive, behavioral, motor, or neuropsychiatric) are common, and sometimes, complex, mixed, and associated with different neuropathology [479,480]. In addition, psychiatric symptoms (apathy, agitation, depression, delusions, etc.) complicate the clinical aspects [481]. This heterogeneity (clinical and biological) is less marked, although also appears in early AD [482,483]. In the 21st century the relevance of previously not well-known brain pathologies (vascular, LBD, TDP-43 (mainly LATE), hippocampal sclerosis, argyrophilic grains), associated with the hallmarks of AD pathology and with other biologic features of aging is notorious (Section 3.2.6) [367,395,420,451,484,485,486]. The AD physiology is also complex and includes inflammation, neuroimmune disturbances (with activation of microglia), altered glucose, cholesterol and lipid metabolism, oxidative stress, chronic hypoperfusion, mitochondrial and energetic dysfunction, neuronal cell cycle reentry, and extra-brain interactions (Section 3.2.6) [487,488,489]. Many reviews emphasize these clinical, pathological, and physiological complexities and describe AD as a clinicopathologic syndrome explicitly or implicitly [144,180,252,447,468,474,475,490,491,492]. In summary, AD has the clinical and biological heterogeneity that occur in syndromic disorders. Obviously, many authors prefer to consider AD as a disease with clinical, physiologic, and pathological heterogeneity—see Table 3 and examples from references [218,493]—usually basic researchers plan and develop their projects under this assumption, and mass media support this idea. 

To envisage the future AD concept, it is reasonable to observe the past. As we demonstrated through this review, AD began as a rare PreSD, according to those who described the first cases, then it was replaced by a wide concept of SDAT by the NIA and its collaborators and presented as the *major killer* in the US during the seventies [56,126,156,157]. Recently, AD was redefined as a biological disease with three pathological hallmarks [183,250] with or without cognitive deterioration. According to these last criteria, in Olmsted County the elderly had three times more biological burden of AD than that of clinical dementia [251]. What does the future hold for this complex concept of AD? Safe to say, the future concept is unpredictable. 

Several authors expressed that the only way to validate a nosological definition of AD would be the development of a relevant effective treatment or a robust animal model. Currently, neither alternative seems feasible. It is unlikely that a magic elixir or a golden bullet cure AD [180,420], nor is a robust animal model of AD on the near horizon. Although a few non-human primates may exhibit neuropathological lesions like those found in AD [494], there are no animals with spontaneous AD to probe drugs [495]; AD is a quasi-unique disorder of *homo sapiens* [320]. 

Is it possible to develop a multi-target drug against multiple AD pathologies? It is possible, but is a tremendous challenge [496]. Although it has been suggested that ‘dirty drugs’ (with multiple actions) could be a solution [497], it is very difficult to treat a single AD pathology (amyloid, tau), and a combined treatment against several associated brain pathologies (AD, vascular lesions, TDP43) seems to be a much more difficult task. The trials against the tau protein and other brain proteinopathies had been unsuccessful up to now [73,283,284,285,498,499]. It is important to recall that the ACH [65,66], the dominant paradigm of AD [86], has been continuously tested over two decades [500,501], and has lost its brilliant longstanding discourse [502] due to the updated information from pathology and physiology, and mainly to the unsuccessful trials. It is currently untenable that *AD is amyloid* (Section 3.2.6) [49,138,232,233,275,276,277,278,279,341,342,343,345,354,355,362,503,504]. The new AAbmA (aducanebad, lecanemab, and donanemab) have produced positive small effects and determined some expectancies [205,206], as well as the evidence that these drugs cannot cure AD nor markedly slow its progression. In fact, novel agents addressing non-amyloid, non-tau targets in AD comprise 70% of the agents currently in clinical trials [505].

A very old dilemma [30,54] has reemerged based on the evidence that the majority (near three-quarters) of centenarians become demented [506,507]: Is elderly dementia/AD a unique disorder, or is it a matter of the inevitable aging and death? Many scientists argue that aging is the driver, albeit poorly understood, of elderly dementia/AD, (see Section 3.2.6, Figure 6) [367,395,420,451,484,485,486,503]. For optimistic people, both aging and dementia will be preventable by means of increasing CR and resilience to brain lesions, controlling comorbidities such as inflammation, diabetes, and metabolic disorders, detecting the earliest markers and symptoms of neurodegeneration with innovative diagnostics, and developing multifactorial therapies and precision medicine [496,497,508]. These plans and the newly recovered research in aging are welcome [509,510,511]. It is always possible to find out novel therapeutic approaches in new genetic, epigenetic, proteomic, or immunologic discoveries; health technology can provide innovative diagnostic approaches, such as plasma and digital biomarkers or retina scan, and support new precision medicine with more adjusted treatments personalized for every subject. Finally, we can always trust on the appearance of new ideas or…even on serendipity.

We must stress that we do not know the initial trigger of brain lesions in AD: amyloid derangement and a secondary tau disorder according to ACH [368,369,503], tau according to histology [63,222,223], or others (neuron disruption by calcium, autoimmunity, infectious, parainfectious or other neuroinflammation, neural senescence, etc. [316,317,318,319,496,497,508]). From a historical point of view, AD has been reconceptualized several times and probably still needs a reconsideration. DA Drachman [504] stated this need in the previous decade. Many authors support this reconceptualization [41,49,138,143,270,273,512] for scientific (possible new drugs), ethical (only trials with real possibilities of success), and social reasons (personal and family aids), but it is difficult to state what the best way would be to realize this new conceptualization. 

Meanwhile, individualized preventive actions (such as the FINGER trial and its future activities [49,143,185,270,273]), along with public health prevention [36,41,49,430,431,432,433,434,513] are strategies to reduce the incidence of dementia/AD as recommended by WHO [184]. There is another open question: Is it possible that the decrease in rich countries of dementia/AD would be continuous along the time? The way of promoting dementia/AD prevention probably would produce positive effects on its incidence, analogous to those observed with preventive actions in cerebrovascular disorders [514,515]; but it is better to be cautious because some RF (obesity, sedentary lifestyle) may have a future negative impact in the developed countries [516].

A clear priority is to invest in the care of demented people [36,49,110,152,185,270,517], promoting dignity to these persons with community and social actions [36,41,49,110,152,154,185,427,517]. Such an approach holds a promising future.

## 4. Conclusions

This review analyzes the complex clinical and pathological evolution of AD over a span of more than 100 years. An eminent psychiatrist, Emil Kraepelin, created the concept of AD in the beginning of the 20th century after the description by Alois Alzheimer (1910) of the NFT lesions in the brain of a female with presenile dementia. Kraepelin’s arbitrary decision based on only four cases of this disorder (some of them atypical according to the current criteria), was considered inadequate by many neuropathologists at that time and have sparked and extensive subsequent debate.

During more than 50 years, AD was a rare disorder. However, in the late seventies, it became the most prevalent form of dementia due to the contribution of three brilliant USA scientists: Terry, a pathologist; Katzman, neurologist; and Butler, a psychiatrist, within only two decades. They developed an intelligent discourse accepted by the American establishment, NIA, society, and mass media. They pointed out that presenile and senile dementia with the AD pathologic hallmark were the same disease (SDAT), contradicting Kraepelin. In addition, they sustained that this disorder was not the consequence of aging (senility), or brain arteriosclerosis, but a different disease, which could be cured if adequate medical research was developed and applied. In the nineties, with the arrival of the genetic revolution, the main cause of AD was attributed by geneticists (Hardy and collaborators) to the deposit in the brain of β-amyloid; this is still the common paradigm of this disorder. The unsuccessful outcomes of numerous trials with several drugs able to eliminate this protein from the patients’ brains have casted doubt on this hypothesis. Over a hundred other hypotheses have been proposed, and many corresponding therapies have also yielded negative results, except for some drugs, acting on the neurotransmitter brain systems (see Table 2), and recently some monoclonal antibodies against amyloid that generate a small benefit.

In the 21st century, AD has been redefined as a biological entity based only in biomarkers (amyloid and tau) for research purposes and to facilitate the development of new therapies, mainly in mild or preclinical (MCI) stages. We are still expecting the successful consequences of this approach. Emerging evidence suggest that AD is more heterogeneous than previously thought (the classical pathologic hallmarks, amyloid and tau, were not the most frequent findings in the brain of the oldest old) has supported the opinion that AD may be a syndrome and not a disease. Finally, the good news: in rich countries, AD and elderly dementia incidence has been decreasing in the last 20 to 30 years, perhaps due to better education and improved lifestyles.

In this intriguing landscape, many authors demand a reconceptualization of AD. They sustain that AD is not only a matter of amyloid or tau brain deposition; it has other multiple derangements (immunity, oxidative stress, inflammation, metabolic and vascular disturbances, and others) and it is clearly entangled with aging. It is difficult to establish new therapies without a better knowledge of its physiopathology and many authors agree on the inexistence of a magic elixir or golden bullet drug to cure this disorder. At this time, it seems ethical to focus on its prevention and the care of individuals afflicted with this disorder. Even with its controversial past beyond us, the medical future of AD is, at this moment, very difficult to predict.

## Figures and Tables

**Table 1:** Milestones in the Alzheimer disease and SD history.

Year/Author	Milestones
1907/A Alzheimer [11]	Described NFT in presenile dementia
1907/Fischer [18]	Senile plaque (SP) was related to senile dementia
1910/Kraepelin [15]	Created Alzheimer disease (AD)
1936/Rothschild [75]	Introduced the personal factors in dementia *
1948/Newton [54]	The pathology of senile dementia and AD is similar
1962/Kral [158]	Introduced the concept of benign and malignant forgetfulness
1963/Kidd [107]	Discovered paired helical filaments in NFT
1964/Terry et al. [108]	Described ultrastructure of NFT and SP (amyloid)
1967/Roth et al. [113]	AD pathologic diagnosis by means of SP and tau quantification
1972/Giurgea [159]	Nootropic (cognitive enhancers) and dementia treatment
1974/Drachman et al. [160]	Deficit in the cholinergic system and human memory
1975/Butler [120]	Was nominated as the first President of the NIA
1976/Katzman [126]	AD as a *major killer* in USA
1978/Katzman et al. [20]	AD and senile dementia as a unique disease **
1984/Glenner et al. [161]	Purification of amyloid protein
1984/McKhann [162]	Probabilistic clinic-pathologic AD diagnosis
1986/Summers et al. [163]	Tetrahydroaminoacridine (tacrine) for AD
1987/Kang et al. [164]	The APP structure was cloned
1987/St George-Hyslop [165]	Familiar AD maps on chromosome 21
1991/Wirak et al. [166]	The APP transgenic mice expressed amyloid
1991/Hardy and Allops [65]	Amyloid as a central event in AD etiology
1992/Hardy and Higgins [66]	Amyloid cascade hypothesis
1993/Corder et al. [167]	APOE4 is a major risk factor for AD
1995/Braak and Braak [168]	AD staging according to neurofibrillary changes
1996/Rogers et al. [169]	Donepezil for AD
1999/Petersen et al. [170]	Introduction of the MCI concept
2000/AAssociation [24,36,171]	Lobbying strategy in Washington. First Congress.
2002/Elan vaccine [172]	First active vaccine anti-amyloid in humans is halted
2004/Haan and Wallace [173]	Dementia prevention. First review
2007/Schneider et al. [174]	Mixed pathologies are the most frequent in old people dementia
2008/Bertram et al. [175]	Sporadic AD has a complex genetic background
2010/Chouliaras et al. [176]	Epigenetic changes have a role in AD
2010/Olazarán et al. [177]	Nonpharmacological therapies in AD
2013/Boyle et al. [178]	Most of the cognitive decline in old life is not due to AD pathology
2014/Doody et al. [179]	Bapineuzumab and solaneuzumab phase III trials failed in AD
2015/Kachaturian et al. [143,180]	Prevention of AD syndrome
2016/Killin et al. [181]	Environmental risk in AD
2016/Satizabal et al. [182]	Dementia risk reduction in the last three decades (Framingham)
2017/Jack et al. [183]	Biological definition of AD
2019/WHO [184]	Guidelines for risk reduction in cognitive decline and dementia
2020/Livingston et al. [185]	Lancet Commision for Dementia prevention, intervention and care
2021/Richard et al. [186]	Bayesian null hypothesis in amyloid therapy in AD
2022–23/Several authors	Efficacy of Aducanumub and Lecanemub in AD
2023/Lau et al. [187]	Aging and AD

Abbreviations: AA—Alzheimer’s Association; AChEI—Acetylcholinesterase inhibitors; MCI—Mild cognitive impairment; NFT—neurofibrillary tangles. * SP are not matched with dementia many times; ** AD Workshop-Conference approval.

**Table 4:** Selected Alzheimer dementia causal hypotheses.

Causal Hypothesis	Author/Year	Medline References *
Amyloid	Hardy et al. 1992 [65,66,279]	>4100
Aging	Several authors [187,280,281,282]	>2300
Tau	Braak and Braak/1995 [168,283,284,285]	>1430
Synaptic	Terry/1996, 2000 [48,62,286]	>800
Cholinergic	Davies and Maloney/1976 [287,288,289]	>780
Inflammatory	Latta/2014 [290,291,292,293]	>700
Oxidative Stress	Markesbery/1997 [294,295]	>690
Mitochondrial	Swerdlow/2004 [296,297,298]	>475
Mitochondria-associated membranes	Several authors [299,300]	17
Calcium hypothesis	Kachaturian/1994 [140,301]	>410
Vascular dysfunction	Several authors [302,303,304,305,306,307]	>300

**Less frequent** (but with adequate fundaments)		
Two-hit	Zhu/2004 [308,309]	>20
Infections	Itzhaki/2020 [310]	>375
Virus	Ball/1987 [312,313]	>185
Dendritic hypothesis	Cochan/2014 [314,315]	>165
Immunological	Several [316,317,318,319]	>125
Phylogenetic	Rapoport/1989 [320]	>17
Aluminum	Exley/2005 [321]	>88
Myelin (breakdown)	Bartzokis/2004 [322,323]	>90

Summarized from Google Scholar (March 2023) among more than 100 hypotheses. Selected from its initial description, from the top Medline best match citations, or the most recent publication. * Number of Medline references obtained by writing the mesh: Alzheimer disease hypothesis and … (Many citations are included in several hypotheses).

**Table 5:** Selected studies showing dementia/AD decrease in 21st century †.

Author/Year/Country	F-Up #	Dem/Type	Prev	Inc	Study Name
Manton/2005/US [372] **	18	Mixed	↓	-	LNLTCS
Lobo/2007/Spain [373]	>10	Dem	↓	- ***	ZARADEMP
Langa/2008/US [374]	9	Dem	↓	-	HRS
Rocca/2011/US [375] **	10–20	Dem/AD	↓	↓ ^&^	Several
Schrijvers/2012/Holland [376]	10	Dem	-	↓ ^&^	Rotterdam
Satizabal/2016//US [182]	30	Dem	-	↓	Framingham Heart
Matthews/2016/UK [377]	7–12	Dem	-	↓	CFAS
Grasset/2016/France [378]	10	Dem	-	↓ *$	PAQUID-Three City
Ahmadi/2017/UK [379] **	10	Dem	-	↓	ELSA
Derby/2017/US [380]	22	Dem	-	↓	IIDP
Noble/2017/US [381]	7	Dem	-	↓	Einstein Aging
Hendrie/2018/US [382]	9	Dem/AD	-	↓	WHICAP
Seblova/2018/Sweden [383] **	30	Dem	-	↓	National Swedish Registry
Sullivan/2019/2-US [384]	40	Dem	-	↓	Monongahela Valley
Wolters/2020/US-Eur [385]	27	Dem/AD	-	↓	Several
Farina/2022/US [386] **	16	Dem/AD	↓	↓	HRS

† The limited negative studies are not presented to highlight the positive results. Table taken from Bermejo-Pareja et al. [387] and updated. **#** F-up—follow-up in years; * Cohort study; ** Study from database, not from true cohorts; *** Only in men; *$ Only in women; ^&^ Without statistical significance ↓—decreased values. Abbreviations: Prev—prevalence; Inc—incidence; Dem—Dementia; Eur—Europe; UK—United Kingdom. CFAS—cognitive function and ageing; ELSA—English Longitudinal Study of Ageing; HRS—Health and Retirement Study; IIDP—Indianapolis-Ibadan project; LNLTCS—national long term care surveys; WHICAP—Washington Heights-Inwood Columbia Aging; ZARADEMP—Dementia Prevalence Study from Zaragossa, Spain.

**Table 6:** Main risk and protection factors for Dementia/AD †.

Risk Factors for Dementia/AD	Evidence
**Perinatal and early life**	
-Undernutrition	+
-Familial and socioeconomic status factors	+
**Childhood**	
-Dyslexia or language difficulties	+/−
-Low education (Mainly alliteration)	++
-Low socioeconomic status	+
**Adulthood**	
-Low education	++
-Tobacco	++
-Traumatic brain injury	++
-Sedentary habit	++
-High blood pressure (and cardiovascular RF)	++
-Obesity	++
-Diabetes (higher in Asians)	++
-Depression	+/− (could be circular)
-Comorbidity/bad health	+
**Elderly**	
-Frailty (in oldest old)	+
-Social isolation	+
**Others**	
-Hearing deficits (not corrected)	+
-Air pollution	+/−
**Protection factors (PFs)**	
-Physical activity	++
-Mental activity along the life	+
-Healthy diet	+/−
-Fish intake	+/−
-Coffee and the drinks	+

† Synthesis of the data from many authors (quoted in alphabetical order [185,396,397,398,399,400,401,402,403,404,405,406,407,408,409,410,411,412,413,414,415,416]; see the text). Evidence: ++: Strong; +: Moderate-mild; +/−: Insufficient data.

**Table 7:** Relevant clinic-pathological studies in the oldest old †.

Cohort	Site	Age	N	Author/Year
CFAS Study &	UK, 6 sites	All ages	456	Savva/2009 [457]
90+ *	California	M = 97	219	Corrada/2012 [458,459]
ROSMAP **	Chicago	M = 80	1167	Boyle/2013,2021 [178,451]
Several USA $	Various	Centenarians	77	Neltner/2016 [442]
Several International $$	6 cohorts	M = 80	4354	Nicols/2023 [449]

† Only cohorts are reported in the table. The Hisayama cohort from Japan [461] and the AD Centers cohort from USA [462] are not included because they were based on medical data. To simplify the table the cohort titles are not explained, see references. Abbreviations N—number. Main results: & Neocortical brain atrophy is relevant; there is attenuation of dementia pathology after 75 years of age. * 22% of demented cases without neuropathological findings; similar prevalence of microinfarcts and NFT; most frequent: mixed dementia (vascular = AD = primary age related tauopathy). ** Mixed pathology predominant (230-pathology combinations). AD pathology explained dementia (only 50%) at individual level. $: all cases had primary age related tauopathy. $$: 12 lesions were studied. Multimorbidity in 91%. Vascular lesions were inconsistently evaluated.

## Abbreviations

AA	Alzheimer’s Association (in USA)
AAbmA	anti-amyloid-beta monoclonal antibody
AchEI	acetylcholinesterase inhibitor
ACH	amyloid cascade hypothesis
AD	Alzheimer disease
ADAD	autosomal dominant AD
ADNI	Alzheimer’s Disease Neuroimaging Initiative
ADRDA	Alzheimer’s Disease and Related Disorders Association
Amyloid	amyloid beta peptide; beta-amyloid; Aβ, are synonymous
ApoE4	apolipoprotein E4
APP	amyloid precursor protein
CR	cognitive reserve
CSF	cerebrospinal fluid
FDA	Food and Drug Administration (USA)
HS	Hippocampal sclerosis
LATE	Limbic predominant age-related TDP-43 encephalopathy
LBD	Lewy body dementia
MCI	mild cognitive impairment
NIA	National Institute on Aging (USA)
NINCDS	National Institute of Neurological and Communicative Disorders and Stroke (USA)
NIH	National Institute of Health (USA)
NFT	neurofibrillary tangle
PreSD	presenile dementia
PS1 and PS2	presenilin 1 and presenilin 2
RF	risk factor
PF	protective factor
SAD	sporadic AD
SES	socioeconomic status
SD	senile dementia
SDAT	senile dementia of Alzheimer type
SP	senile plaque (including neuritic senile plaque)
TDP-43	TAR DNA-Binding Protein 43
VaD	vascular dementia
WHO	World Health Organization
